# Photothermal Detection of MicroRNA Using a Horseradish Peroxidase-Encapsulated DNA Hydrogel With a Portable Thermometer

**DOI:** 10.3389/fbioe.2021.799370

**Published:** 2021-12-13

**Authors:** Xiujuan Liu, Meixiang Zhang, Ze Chen, Jiuqing Cui, Long Yang, Zihe Lu, Fang Qi, Haixia Wang

**Affiliations:** ^1^ Department of Intensive Care Unit, The First Hospital of Qinhuangdao, Qinhuangdao, China; ^2^ Department of Intensive Care Unit, Hebei Petrochina Central Hospital, Langfang, China; ^3^ Department of Intensive Care Unit, Chengde Medical University, Chengde, China

**Keywords:** detection of MicroRNAs, photothermal detection, biosensor, hydrogel, tumor detection marker

## Abstract

MicroRNA (miRNA) detection has attracted widespread interest as a tumor detection marker. In this work, a miRNA-responsive visual and temperature sensitive probe composed of a horseradish peroxidase (HRP)-encapsulated DNA hydrogel was designed and synthesized. The biosensor converted the miRNA hybridization signal to a photothermal effect which was measured using a digital thermometer. The substrate DNA linker strand of the hydrogel hybridizes with different sequences of miRNA resulting in the collapse of the hydrogel and the release of HRP. HRP oxidizes 3,3′,5,5′-tetramethylbenzidine (TMB) resulting in a color change and a strong photothermal effect was observed after shining near-infrared light on the oxidized product. The thermometer-based readout method has a wide linear range (0.5–4.0 µM) and a limit of detection limit of 7.8 nM which is comparable with traditional UV-vis absorption spectrometry detection and quantitative real time polymerase chain reaction methods. The low cost, ease of operation, and high sensitivity shows that this biosensor has potential for point-of-care biomolecular detection and biomedical applications.

## Introduction

MicroRNAs (miRNAs) are endogenous non-coding RNAs and post-transcriptional gene regulators which are closely related to tumor occurrence and development (Wightman et al., 1993; Bartel, 2009; Lin and Gregory, 2015; Das and Ghosal, 2018). Deregulated miRNAs exist in the plasma and serum of many cancer patients (Lin and Gregory, 2015; Armand-Labit and Pradines, 2017; Ji and Guo, 2019; Si et al., 2020). Increased miRNA expression is correlated with cancer cells or the presence of tumorous tissues and high levels are also found in the peripheral blood (Mitchell et al., 2008; Boeri et al., 2011; Kelly et al., 2013). Thus, elevated miRNA levels are tumor markers and their detection has attracted widespread interest. Detection of miRNA levels over the past decade has included northern blotting, real-time quantitative polymerase chain reaction (qRT-PCR), flow cytometry, microarray, and enzyme-catalyzed amplification technology (Calin et al., 2004; Guo et al., 2009; Git et al., 2010; Pritchard et al., 2012; Porichis et al., 2014). However, high-efficiency and sensitive detection of miRNAs is challenging because they have low abundance, readily degraded, require accurate temperature control, and involve complex processes and expensive equipment (Li et al., 2016). The current gold standard method for detecting miRNA by northern blotting is time consuming, has low sensitivity, is at risk of degradation by RNases, and involves the use of carcinogenic chemicals (ethidium bromide and formaldehyde). Meanwhile, qRT-PCR exhibits highly sensitive detection but it suffers from poor selectivity and low specificity (Li et al., 2020). Alternatively, biosensors based on fluorescence, electrochemistry, photoelectrochemistry, and chemiluminescence are promising analytical technologies with high selectivity and sensitivity compared with conventional miRNA detection methods (Zheng et al., 2019a; Wang et al., 2020; Jin et al., 2021). Biosensing detection based on visual recognition and quantitation through a portable readout is potentially an ideal detection approach because output signals are obtained by simple portable analytical instruments or the naked eye which overcomes the limitations of assay readout methods dependent on complex, expensive, and bulky analytical equipment (Qu et al., 2011; Fu et al., 2016; Zheng et al., 2019b; Zhao et al., 2019; Xu et al., 2020; Xu et al., 2021).

The horseradish peroxidase (HRP)-3,3′,5,5′-tetramethylbenzidine (TMB)-H_2_O_2_ system (HRP-TMB- H_2_O_2_) has been explored as a classical system for portable qualitative detection. HRP catalyzes the one-electron oxidation of TMB to generate a blue colored charge-transfer complex of oxidized TMB (oxTMB) with an absorbance maximum of 652 nm. OxTMB also exhibits a strong near infrared (NIR) laser-driven photothermal effect which could be used as a highly sensitive photothermal probe (Fu et al., 2016). However, HRP is easily affected by the detecting environment. Hydrogel is a type of cross-linked hydrophilic polymer and a large amount of water can be absorbed. Hydrophilic polymers can be dissolved in water without a defined shape, however, after cross-links, the solid-like three-dimensional structures are formed, which bring a uid-like properties (Guan et al., 2020; Wei et al., 2020). Stimulus-responsive (or target-responsive) DNA hydrogels composed of multifunctional polymers as the backbone and functional DNA as the cross-linker are potential colorimetric sensors carrying enzymes because of their biocompatibility, encapsulation and release capability, flexibility, and mechanical stability (Xiang and Lu, 2012; Kahn et al., 2017; Amalfitano et al., 2021; Liu et al., 2021). They are widely used for the determination of various targets including ions, small molecules, nucleic acids, and proteins (Zhu et al., 2010; Lin et al., 2011; Yan et al., 2013).

**SCHEME 1:**
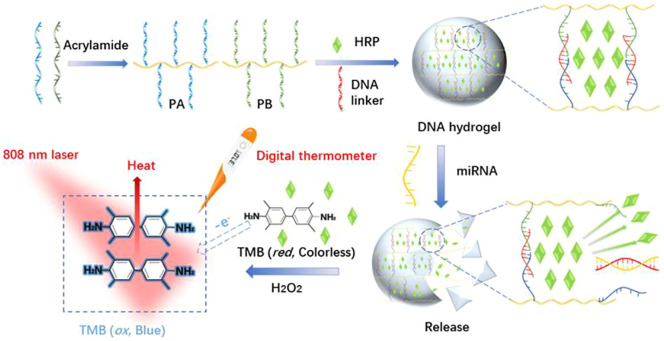
Schematic illustration of the synthesis of stimuli-response DNA hydrogels and photothermal sensing detection of miRNAs based on HRP-Mediated TMB-H_2_O_2_ colorimetric system.

Herein, a target-responsive DNA hydrogel-based biosensor was generated and applied for visual recognition and portable photothermal quantification of miRNAs using a common thermometer readout (Scheme 1). PA and PB were synthesized by copolymerization of acrylic DNA and acrylamide monomers, cross-linked with a substrate DNA linker strand containing partial complementary sequences with PA and PB, and complete complementary sequence of miRNA to form a hydrogel with encapsulated HRP. In the presence of miRNA, the substrate DNA linker strand hybridized with miRNA which led to the collapse of the hydrogel and HRP release. Then, released HRP oxidized TMB-H_2_O_2_ and formed a blue colored product. Laser NIR irradiation of oxidized TMB at 808 nm exhibited a strong photothermal effect resulting in the conversion of the miRNA hybridization signal to heat. A digital thermometer detected the signal with a linear detection range from 0.5 to 4.0 µM and a limit of detection of 7.8 nM. Therefore, this strategy achieved visual recognition and portable photothermal quantitation of miRNAs.

## Materials and Methods

### Materials and Reagents

Acrylamide, ammonium persulfate (APS), and H_2_O_2_ (30%) were obtained from Sinopharm Chemical Reagent (Shanghai, China). 3,3′,5,5′-tetramethylbenzidine (TMB), N,N,N′,N′-tetramethylethylenediamine (TEMED), tris(hydroxymethyl)aminomethane (Tris), Dulbecco’s modified Eagle’s medium (DMEM), fetal bovine serum (FBS), and penicillin-streptomycin were obtained from Sigma-Aldrich (St. Louis, MO, USA). HRP was purchased from J&K Scientific Ltd. (Beijing, China). TRIzol solution, acrylic-DNA and all other oligonucleotides used in this study (Supplementary Table S1) were synthesized by Sangon Biotech Co., Ltd. (Shanghai, China). Other reagents were purchased from Damao Chemical Reagent Factory (Tianjin, China). HeLa cells were obtained from Sangon Biotech Co., Ltd.

### Synthesis of the DNA Functional Linear Polyacrylamide Chains

A typical synthesis involved mixing 10 μL acrylamide (25% w/v) with 20 μL Tris-HCl pH 8.0 (10 mM), followed by the addition of 16 μL acrydite-DNA solution (10 μM SA or SB in Supplementary Table S1). The mixture was kept in a N_2_ atmosphere at 20°C for 10 min to remove air. Next, 2 μL of APS (4% w/v) and 2 μL TEMED (5% v/v) were added, and the solution was incubated in a N_2_ atmosphere for a further 15 min. The resulting functional DNA linear polyacrylamide chains (PA and PB) were stored at 4°C for subsequent use.

### Preparation of the HRP -Encapsulated Stimulus-Responsive Hydrogel

10 μL (10 μM) DNA functional linear polyacrylamide solution, PA and PB, were mixed at room, and then 40 ng HRP (10 μL 4 μg/ml) was added to them. 10 μL (10 μM) DNA (L1) was added into the above mixture was incubated at 37°C for 30 min. After being washed three times with 10 μL wash buffer (containing Tris-HCl (10 mM), NaCl (50 mM), and MgCl_2_ (10 mM), pH 8.0) and followed by lyophilization the HRP-encapsulated stimulus-responsive hydrogel was obtained.

### Detection of miRNA

For the target miRNA assay, 5 μL H_2_O_2_ (0.4 mM) and 10 μL of TMB (0.4 mM) were added to a 0.5 ml tube at room temperature and incubated until the solution was separated into two colorless layers. Then, 10 μL miRNA target with different concentrations was added into the hydrogel-containing tube and incubated for 15 min at 37°C to ensure the complete disassociation reaction. A series of blue solutions was obtained. 5 μL of this solution was taken to measure the absorbance at 650 nm with a UV spectrophotometer. Another 5 μL was taken to investigate the photothermal effect by recording the temperature using a common digital thermometer [Sangon Biotech Co., Ltd. (Shanghai, China)] under 808 nm laser at a power density of 5.26 W cm^−2^ for 300 s.

### miRNA Extraction From HeLa Cells

HeLa cells, were cultured in Dulbecco’s modified Eagle’s medium (DMEM) supplemented with 10% fetal bovine serum (FBS), penicillin (100 units/mL), and streptomycin (100 μg/ml) in an incubator containing (5% CO_2_, 37°C). After the cells were all over the bottom of the bottle, the target miRNA was extracted using TRIzol solution following the instructions and was analyzed by above photothermal biosensor. Moreover, the results were verified by qRT-PCR method.

## Results and Discussion

### Characterization of the HRP -Encapsulated Stimulus-Responsive Hydrogel

**FIGURE 1:**
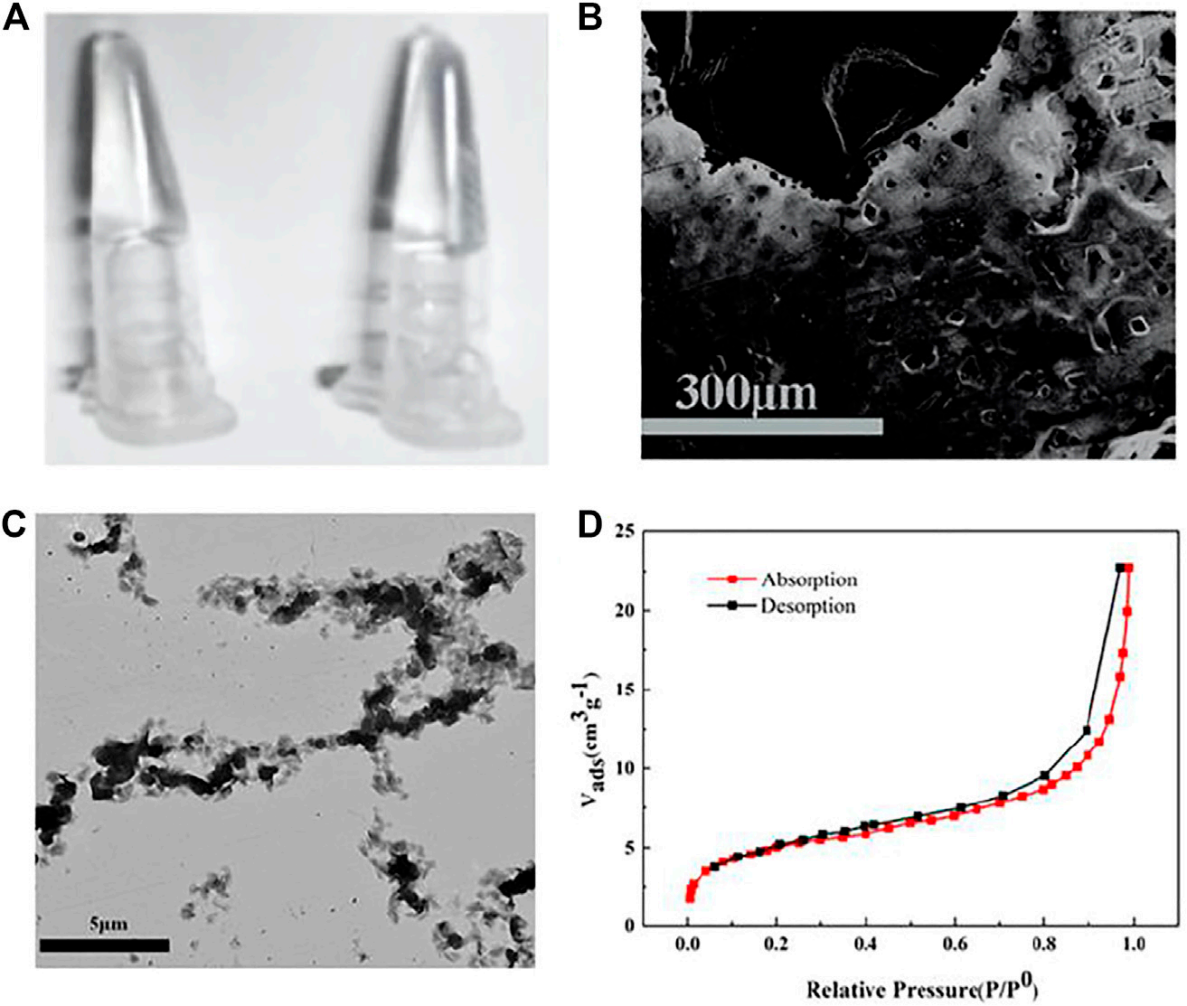
**(A)** Digital photographs, SEM image **(B)**, TEM image **(C)**, N_2_ physisorption isotherm **(D)** of HRP -encapsulated stimulus-responsive hydrogel.

PA and PB synthesized by copolymerization of acrylic DNA and acrylamide monomers were validated by polyacrylamide gel electrophoresis (PAGE, Supplementary Figure S1). The substrate DNA linker strand containing partial complementary sequences with both PA and PB, and complete complementary sequence of miRNA cross-linked with PA and PB formed the HRP-encapsulated stimulus-responsive hydrogel (Figure 1A) which formed a porous, three-dimensional network structure observed by SEM (Figure 1B). A vial inversion test was adopted to show hydrogel formation as Supplementary Figure S2. The element mapping images demonstrated the distribution of Fe, P, and N (Supplementary Figure S3) which provided direct evidence that HRP was trapped in the hydrogels and DNA strands participated in the construction of HRP-encapsulated hydrogels. TEM images indicated that the functional linear DNA polyacrylamide chains were interconnected in HRP-encapsulated hydrogels (Figure 1C). The surface area was calculated as 15.9 m^2^ g^−1^ (Figure 1D) by Brunauer-Emmett-Teller (BET) model (Naderi and Tarleton, 2015).

### Principle and Feasibility of Visual Recognition and Photothermal Quantitation of MicroRNAs Based on Target-Responsive DNA Hydrogels

In the absence of miRNA, HRP was stably trapped inside the hydrogel and physically separated from TMB-H_2_O_2_ which was in the solution outside the hydrogel. After the addition of target miRNA, hybridization of the substrate linker strand with miRNA led to the collapse of the hydrogel and the release of HRP. HRP oxidized TMB-H_2_O_2_ with the one-electron transfer generated in TMB forming the blue-colored, charged, oxidized TMB (oxTMB) complex which generated heat following NIR laser irradiation at 808 nm (Fu et al., 2018). Hence the hybridization signal of miRNA was converted into a photothermal effect.

**FIGURE 2:**
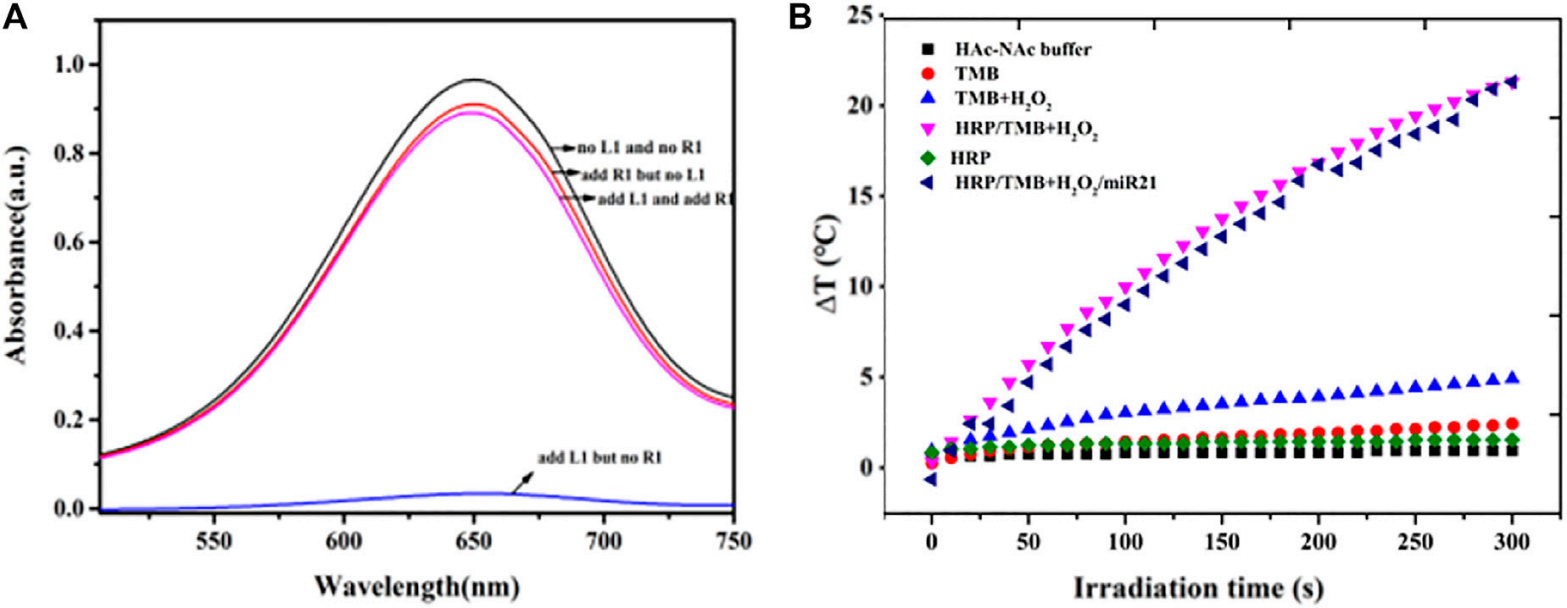
**(A)** Feasibility test of the release of HRP in hydrogels based on the intensity of absorption peak of oxTMB at 650 nm **(B)** Photothermal evolution of different components including HAc-NaAc buffer, TMB, TMB-H_2_O_2_, HRP, HRP-TMB-H_2_O_2_ under 808 nm laser at a power density of 5.26 W cm^−2^ for 300 s.

The intensity of the 650 nm absorption peak representing oxTMB was used to determine the encapsulation and release of HRP in hydrogels (Figure 2A). Weak oxTMB absorption was observed in the presence of crosslinking DNA (L1) and in the absence of miRNA (R1) indicating that most of the HRP was trapped inside the DNA hydrogel. When the crosslinking DNA (L1) and target miRNA (R1) were absent, a stronger absorption peak appeared after TMB-H_2_O_2_ was added (black line) indicating that PA and PB did not trap HRP in the DNA hydrogel. When miRNA (R1) was added, the absorption peak of oxTMB slightly increased (red line) indicating that crosslinking DNA is required to trap HRP inside the DNA hydrogel. This was confirmed by the addition of crosslinking DNA (L1) resulting in decreased absorption indicating that the DNA hydrogel was constructed and some HRP was encapsulated. The addition of TMB-H_2_O_2_ resulted in little absorbance change showing that there were only trace amounts of free HRP in the supernatant. Subsequent addition of target miRNA resulted in a gradual increase in the oxTMB absorption peak demonstrating that HRP was released from the hydrogel (purple line).

The photothermal properties of HAc-NaAc buffer, TMB, TMB-H_2_O_2_, HRP, and HRP-TMB-H_2_O_2_ were determined under 808 nm laser at a power density of 5.26 W cm^−2^ for 300 s to investigate the feasibility of the HRP-catalyzed TMB-H_2_O_2_ system for photothermal conversion (Figure 2B). A dramatic temperature increase appeared in the HRP-TMB-H_2_O_2_ system, while no apparent temperature increases were exhibited in all other cases. Therefore, the HRP-TMB-H_2_O_2_ system based on target-responsive DNA hydrogels is a suitable biosensor to detect miRNA using a thermometer.

### Optimization of Assay Condition for Visual Recognition and Photothermal Quantitation of MicroRNAs

**FIGURE 3:**
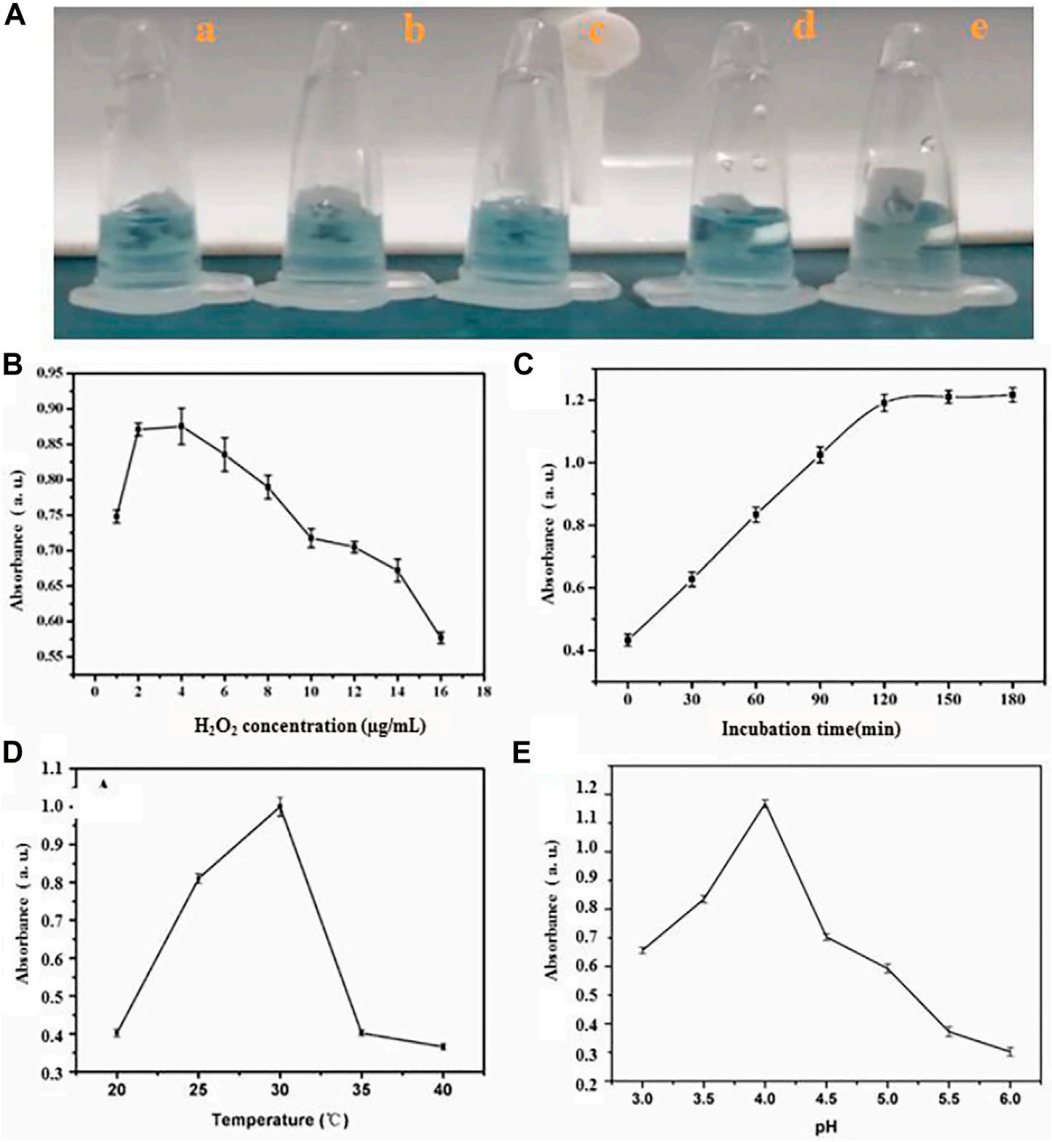
The influence of **(A)** amount of HRP (a) 4 μg/ml (b) 6 μg/ml (c) 8 μg/ml (d) 9 μg/ml (e)10 μg/ml, **(B)** quantity of H_2_O_2_ **(C)** incubating time **(D)** temperature and **(E)** pH value for sensing system.

The optimal incubation time, temperature, amount of HRP and H_2_O_2_ was tested to achieve sensitive detection of miRNA using the HRP-encapsulated DNA hydrogel/TMB-H_2_O_2_ probe (Figure 3). 4 μg/ml HRP was chosen as the optimal loading concentration, since higher concentrations would result in the leakage of enzyme, which would bring the false positives of investigation (Figure 3A). The intensity of oxTMB absorption increased from 1 to 4 nM H_2_O_2_ however, the intensity of the absorption peak sharply decreased beyond 4 nM demonstrating that inhibition of the catalytic reaction occurred (Figure 3B). Therefore, 4 nM H_2_O_2_ was chosen as the optimal concentration for miRNA detection.

The absorption intensity of oxTMB plateaued at 120 min incubation time (Figure 3C) demonstrating that the hybridization process of substrate linker strand with miRNA was complete. Therefore, 120 min was chosen as the optimal incubation time. OxTMB absorption intensity increased from 20°C to 30°C, then stabilized up to 40°C (Figure 3D). Therefore, 30°C was taken as the optimal assay temperature. Finally, a pH of 4.0 was chosen as the ideal condition for maximal oxTMB absorption intensity (Figure 3E).

### Performance of Biosensor Based on HRP Encapsulated DNA Hydrogels/TMB- H_2_O_2_ System for miRNA Determination

**FIGURE 4:**
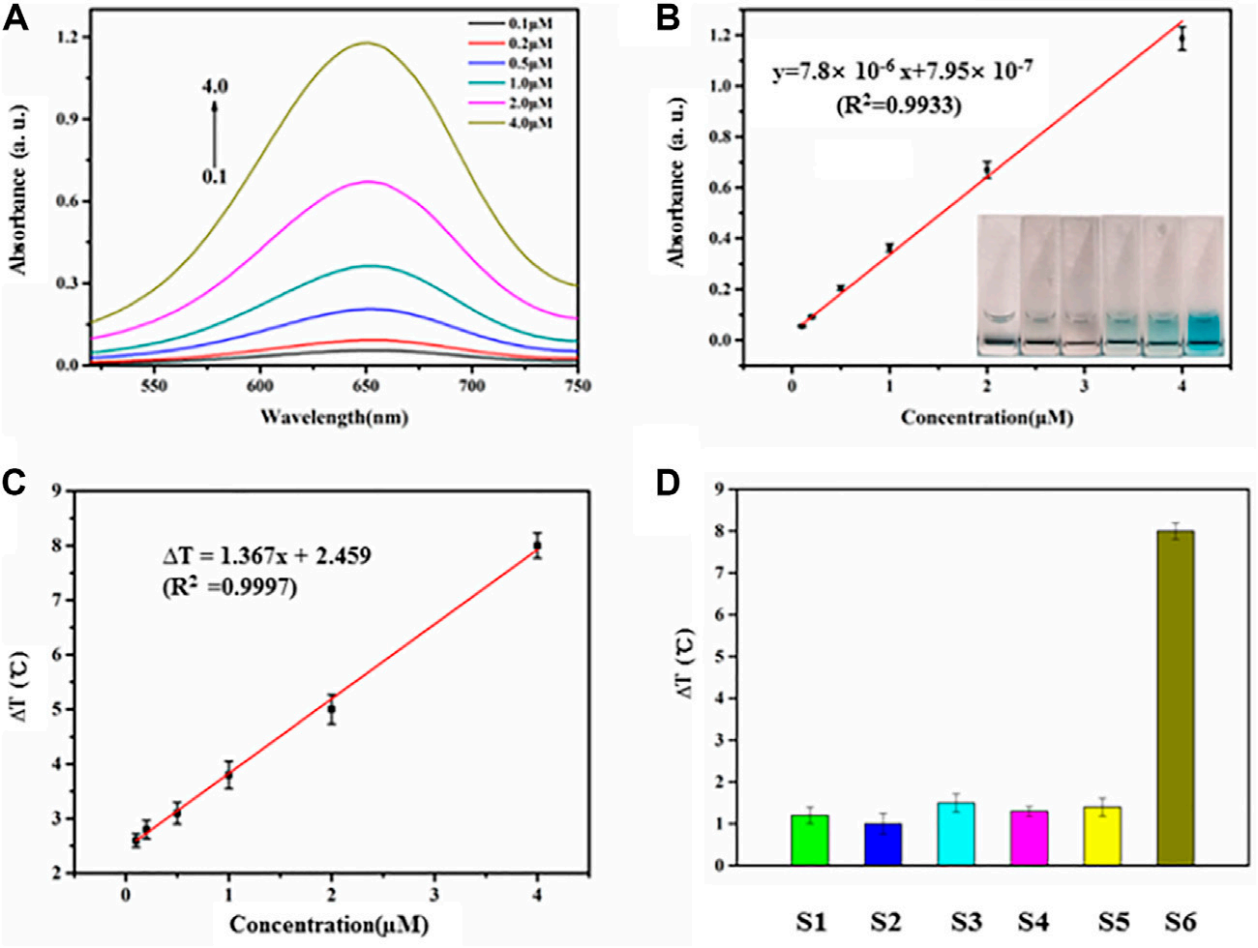
**(A)** Absorbance intensities at 650 nm were obtained upon addition of miR21 with different concentrations (0.1, 0.2, 0.5, 1.0,2.0, 4.0 µM). **(B)** The relationship of absorbance intensities and miRNA concentration. The range of miR21 concentration is from 0 to 4.0 µM. **(C)** The relationship of temperature evolution and miRNA concentration from 0.5 to 4.0 µM. **(D)** Investigation of the selectivity of the miRNA-responsive HRP encapsulated DNA hydrogels/TMB- H_2_O_2_ (S1, miR18; S2, miR205; S3, miR141; S4, miR25; S5, miR183; S6, miR21).

The biosensor exhibited a concentration-dependent effect following the addition of increasing amounts of miR-21 extracted from HeLa cells under the optimized conditions (Figure 4A). The transition from colorless to blue (Figure 4B, inset) is easily distinguished by the naked eye. There is a linear relationship between the intensity of the absorption peaks at 650 nm and miRNA concentration between 0 and 4 µM (Figure 4B). The regression equation is y = 7.8 × 10^−6^ x +7.95 × 10^−7^ (R^2^ = 0.9933), where x is miR-21 concentration; y is the intensity of absorption peaks at 650 nm; R^2^ is the correlation coefficient. The detection limit was approximately 10 nM (signal-to-noise ratio = 3). Similarly, the temperature increase following NIR illumination was linearly proportional to the miR21 concentration between 0.5–4.0 µM when the irradiation time was 90 s (Figure 4C, Supplementary Figure S4). The regression equation was ∆T = 1.367x + 2.459 (R^2^ = 0.9997), where x is the miRNA concentration, ∆T is the temperature increase, and R^2^ is the correlation coefficient. The detection limit was approximately 7.8 nM (signal-to-noise ratio = 3). Six experiments were carried out at 2 ng ml^−1^ miR-21, and the relative standard deviation was 5.2% over a 5-week period showing that the biosensor results were stable and reproducible. Moreover, other miRNAs with different sequences were similarly detected by the biosensor due to the precise base pairing (Figure 4D).

### Detection of miRNA in Cell Lysates

**FIGURE 5:**
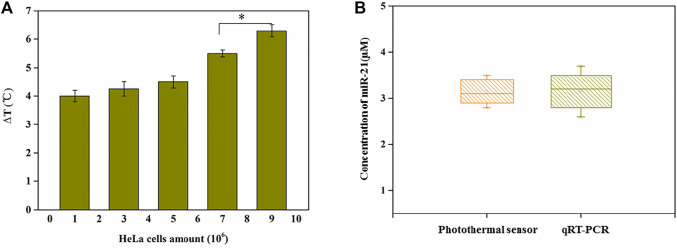
**(A)** Temperature changes with the endogenous miR21 extracted from different amounts of HeLa cells. An asterisk indicates a statistically significant difference (*p < 0.05). **(B)** Detection of miR-21 using current photothermal sensor and qRT-PCR from HeLa cells (concentration: 9 × 10^6^).

As the detection for miRNA above, the proposed photothermal sensor based on thermometer-based readout composed of miRNA -responsive HRP encapsulated DNA hydrogels/TMB-H_2_O_2_ showed high selectivity, sensitivity and reproducibility. We detected miR-21, which was extracted from HeLa cells using TRIzol, through this biosensor to further evaluate its feasibility in real biological samples. As we expected, as the amount of HeLa cells increased as Figure 5A shown in, the temperature increased accordingly. Moreover, when the detection of miR-21 in cell lysates of HeLa was performed through this photothermal biosensor, a standard qRT-PCR detection was also carried out simultaneously to verify this. No obvious difference in detecting miR-21 in them as shown in Figure 5B which indicated the effectiveness of this biosensor using for miRNA detection in real biological samples.

## Conclusion

In summary, we designed and synthesized a novel and simple photothermal detection method of miRNA using a common thermometer based on a target-responsive HRP encapsulated DNA hydrogel/TMB-H_2_O_2_ biosensor. The dissociation of the hydrogel was directly controlled by miRNA, and the released HRP catalyzed the TMB-H_2_O_2_ system to form oxTMB which exhibited photothermal properties under 808 nm laser irradiation. The resulting temperature evolution was proportional to the miRNA concentration. The limit of detection was 7.8 nM and the linear range was between 0.5–4.0 µM. Moreover, compared with traditional spectrophotometric or RT-qPCR methods, this method exhibited the distinct advantage including no need for specialized equipment and reagents, high selectivity and specificity of ease of use and greatly reduced cost since there is no need for specialized equipment and reagents. This biosensor method which showed great potential for point-of-care miRNA detection and biomedical applications.

## Data Availability

The original contributions presented in the study are included in the article/Supplementary Material, further inquiries can be directed to the corresponding author.
