# Supplementary material for: Photothermal Detection of MicroRNA Using a Horseradish Peroxidase-Encapsulated DNA Hydrogel With a Portable Thermometer
Source: Front Bioeng Biotechnol. 2021 Dec 13;9:799370. doi: 10.3389/fbioe.2021.799370 (PMC8710733; doi:10.3389/fbioe.2021.799370)
Supplement: Supplementary file 1 [file DataSheet1.docx]

Supplementary Material

# Supplementary Figures and Tables

## Supplementary Table

# Table S1 Acrylic-DNA and all other oligonucleotides sequences (5′−3′)

| miR-21: UAGCUUAUCAGACUGAUGUUGA |
| --- |
| Acrydite-modified DNA (SA): ATGTGTGACTACAACT |
| Acrydite-modified DNA (SB): CTGTTTGTGATAAGCTA |
| miR-18: UAAGGUGCAUCUAGUGCAGAUAG |
| miR-205: UCCUUCAUUCCACCGGAGUCUG |
| miR-141: UAACACUGUCUGGUAAAGAUGG |
| miR-25: CAUUGCACUUGUCUCGGUCUGA |
| miR-183: UAUGGCACUGGUAGAAUUCACU |

## Supplementary Figures

**
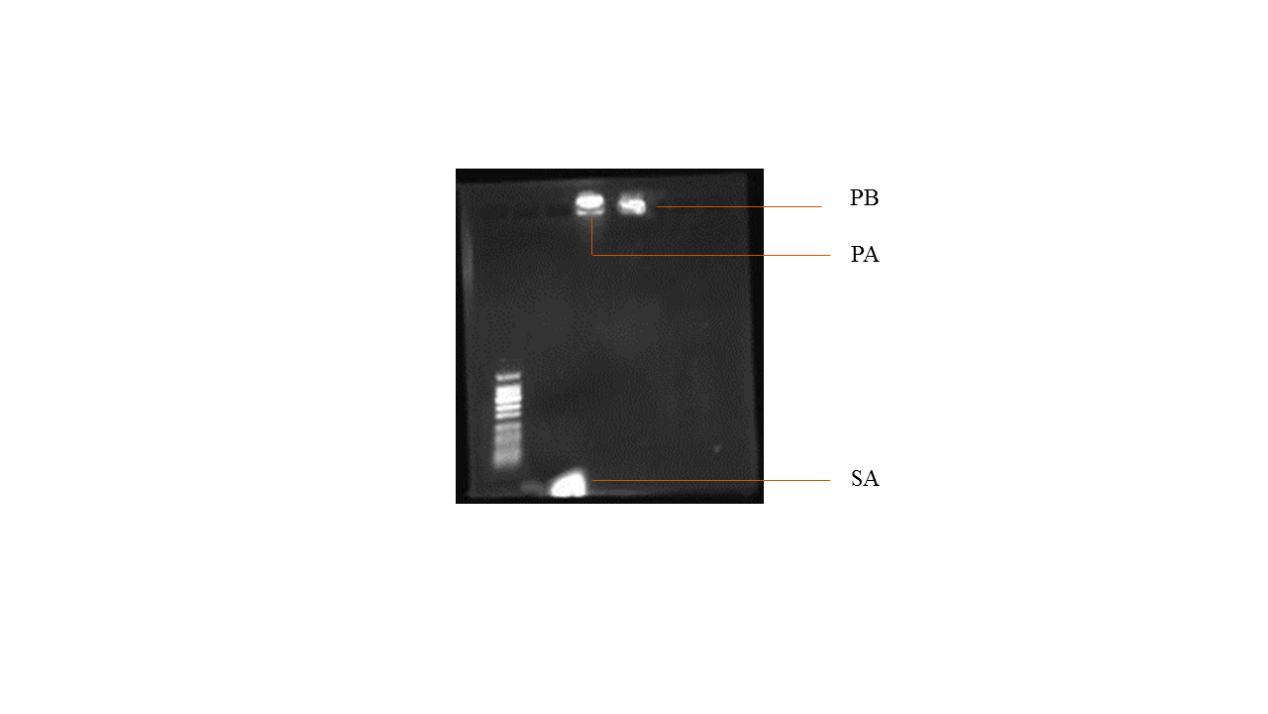
**

**Figure S1** PAGE of Polyacrylamide and acrylamide monomers


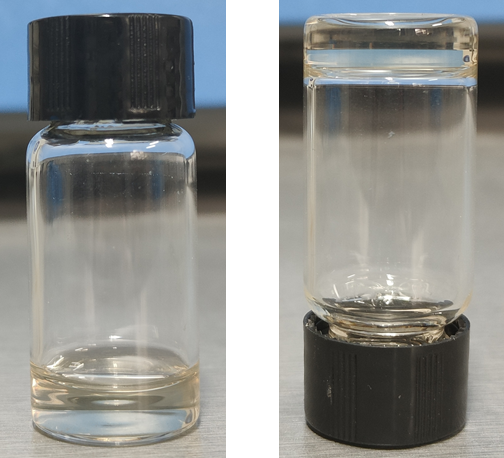


**Figure S2** the vial inversion test of hydrogel formation


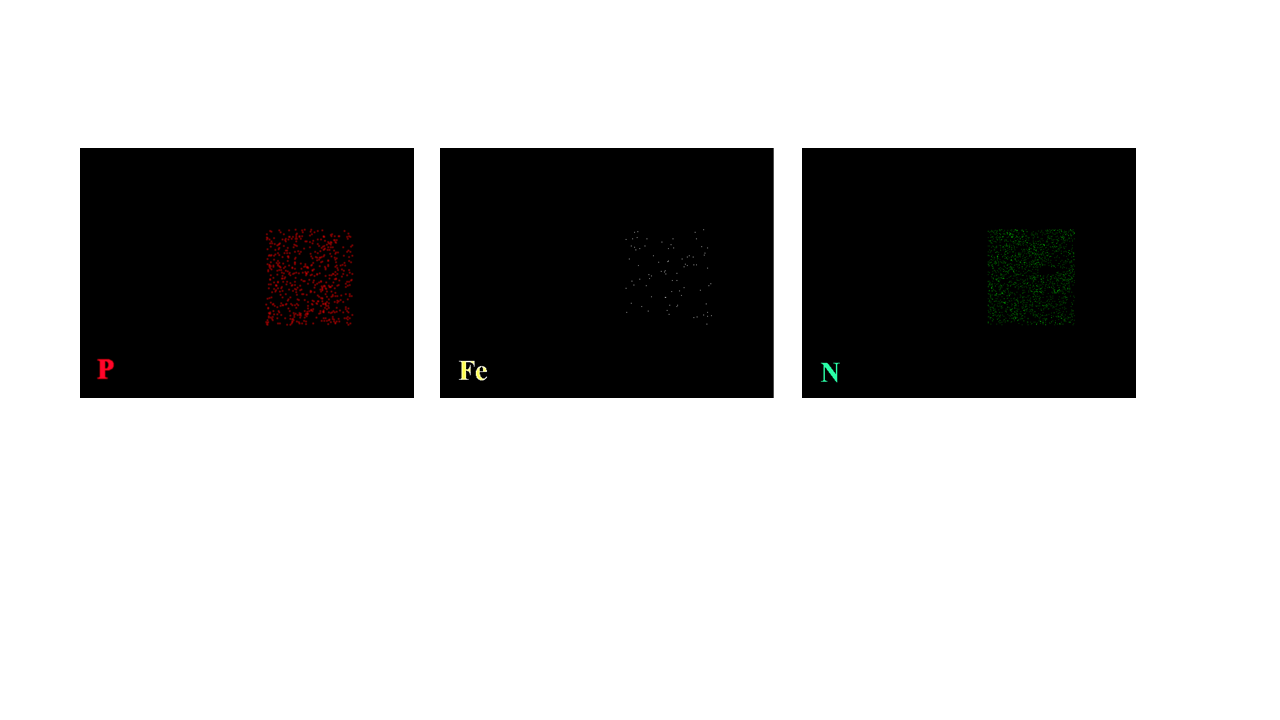


**Figure S3** Element mapping images of HRP-encapsulated hydrogels


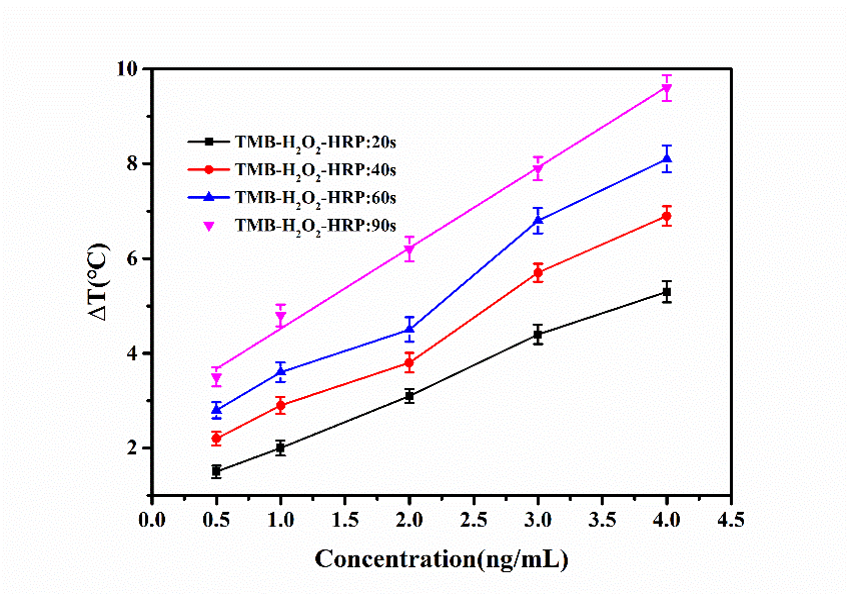


**Figure S4** Temperature evolution under different miRNA concentration and irradiation time
